# Small RNA profiling reveals the involvement of microRNA-mediated gene regulation in response to symbiosis in raspberry

**DOI:** 10.3389/fmicb.2022.1082494

**Published:** 2022-12-21

**Authors:** Zhiyu Yang, Lianmei Yuan, Haifeng Zhu, Jing Jiang, Hongyi Yang, Lili Li

**Affiliations:** ^1^Key Laboratory of Saline-alkali Vegetation Ecology Restoration (Northeast Forestry University), Ministry of Education, Harbin, China; ^2^College of Life Science, Northeast Forestry University, Harbin, China; ^3^Institute of Forestry Science of Heilongjiang Province, Harbin, China

**Keywords:** dark septate endophytes, miRNA, symbiosis, raspberry, post-transcriptional regulation

## Abstract

Dark septate endophytes (DSEs) can form reciprocal symbioses with most terrestrial plants, providing them with mineral nutrients in exchange for photosynthetic products. Although the mechanism of plant-DSEs is well understood at the transcriptional level, little is known about their post-transcriptional regulation, and microRNAs (miRNAs) for the symbiotic process of DSE infestation of raspberry have not been identified. In this study, we comprehensively identified the miRNAs of DSE-infested raspberry symbiosis using Illumina sequencing. A total of 361 known miRNAs and 95 novel miRNAs were identified in the roots. Similar to other dicotyledons, most of the identified raspberry miRNAs were 21 nt in length. Thirty-seven miRNAs were differentially expressed during colonization after inoculation with *Phialocephala fortinii* F5, suggesting a possible role for these miRNAs in the symbiotic process. Notably, two miRNAs (miR171h and miR396) previously reported to be responsive to symbiotic processes in alfalfa also had altered expression during raspberry symbiosis. Gene Ontology (GO) and Kyoto Encyclopedia of Genes and Genomes (KEGG) analyses suggests that miRNAs are mainly involved in regulatory mechanisms, such as biological processes, cellular metabolic processes, biosynthesis of secondary metabolites, plant–pathogen interactions, and phytohormone signaling pathways. This study revealed the potential conservation of miRNA-mediated post-transcriptional regulation in symbiotic processes among plants and provides some novel miRNAs for understanding the regulatory mechanisms of DSE–raspberry symbiosis.

## Introduction

Dark septate endophytes (DSEs) are dominant endophytic fungal groups that colonize plant roots and are mainly characterized by dark-colored hyphae with distinct transverse septa that colonize the epidermis, cortex, or intercellular spaces of healthy plant roots, with few developing pathological features similar to pathogenic fungi within the root tissue (Hou et al., 2020). DSEs have similar functions to mycorrhizal fungi and enable reciprocal symbiosis with host plants (Fracchia et al., 2011; Monica et al., 2015). DSEs contribute to the mineralization of insoluble organic and inorganic phosphorus, promote the uptake and utilization of soil nutrients, such as nitrogen and phosphorus, and enhance plant stress resistance (Yan et al., 2011; Khastini et al., 2012). DSEs have colonization advantages over arbuscular mycorrhiza fungi (AMF) in extreme environments, such as alpine and polar regions, and gradually replace mycorrhizal fungi and perform diverse ecological functions (Jumpponen and Trappe, 1998; Mandyam and Jumpponen, 2005). However, the mechanism of the interaction between DSEs and host plants is not as clear as that of AMF. Therefore, further research is needed to improve plant growth and quality with DSEs.

Plant microRNAs (miRNAs) are a class of endogenous non-coding small molecule RNAs of 18–25 nt in length that are involved in regulating the expression of various genes during plant growth, development, and metabolism (Kurihara and Watanabe, 2004). Recently, the discovery of small RNA sheds light on post-transcriptional gene regulation. High-throughput sequencing has been widely used to identify conserved and novel miRNAs in plants, which has enlarged the realm of miRNA research (Simsek et al., 2017; Jiu et al., 2019). Through small RNA sequencing technology, some known miRNAs and novel miRNAs in non-model plants, such as grass pea (Bhat et al., 2020), cardamom (Anjali et al., 2019), white lupin (Zhu et al., 2010), and Brazilian pine (Galdino et al., 2019), have been successfully identified or predicted. Studies have confirmed that miRNAs play vital roles in plant–microbe symbioses. miR171h has been found to regulate arbuscular mycorrhiza fungi colonization of medicinal plants by targeting NSP2. Studies have also proposed that miRNAs play a role in the arbuscular mycorrhizal signal transduction of tomato by analyzing miRNA expression patterns (Gu et al., 2010; Lauressergues et al., 2012). In addition, Chien et al. found that miRNA mycorrhizal signal transduction is related to nutrient transport, among which miR156 can affect the regulatory pathway of strigolactone in symbiosis formation, and miR171, miR398, miR399, and miR408 play a role in the regulation of phosphorus homeostasis (Chen et al., 2015; Chien et al., 2017; Hussain et al., 2018). Scholars have a preliminary understanding of the regulatory mechanism of miRNA involved in the formation and function of symbioses, but more studies are still at the basic stage of mining and identifying symbiosis-related miRNA and the change in expression level. Therefore, functional studies of miRNA in the symbiosis process still need to be carried out.

Raspberry (*Rubus idaeus* L.) is a shrub with special characteristics in cold regions (Kim et al., 2016). Raspberry leaves are rich in trace elements, gallic acid, salicylic acid, and many other active substances with health functions (Djordjević et al., 2015). Its fruits have been proven to have anti-inflammatory, antioxidant, anti-cancer, and antibacterial functions (Jung et al., 2015; Akkari et al., 2016). In recent years, raspberries are popular as emerging small berries, and the planting area is expanding (Carew et al., 2015; Lebedev et al., 2018). We previously examined miRNAs in raspberry root, stem and leaf tissues using high-throughput sequencing technology to further elucidate the role of miRNAs in raspberry growth and development (Yan et al., 2020). Similar to other plants, raspberries recruit fungi to their roots as they grow, thus facilitating the host’s uptake of water and minerals from the soil, which in turn promotes plant growth (Oszust and Frc, 2020).

At present, most studies on the molecular mechanisms of symbiotic miRNAs are based on herbaceous and woody model plants (Zhu et al., 2010; Anjali et al., 2019). The mechanism of action of symbiotic miRNAs in Rosaceae is still unclear, and a study of raspberry and symbiosis-related miRNAs has not been reported. The formation of symbiosis is a dynamic and complex regulatory process, and after mycelial invasion, plant cells undergo a complex reconstruction process, leading to a series of complex morphological and physiological changes. Based on the important regulatory role of miRNAs in plant growth and development, this study identified raspberry-DSE symbiosis-related miRNAs and target genes influencing plant performance traits by regulating the expression levels of related miRNAs in plants to screen miRNAs that positively regulate the symbiosis formation process and promote the effective colonization of raspberry roots by fungi and their proliferation.

## Materials and methods

### Dark septate endophytes and plant materials

*Phialocephala fortinii* F5 was stored at −4°C on potato dextrose agar (PDA, containing 200 g/l^−1^ potato extract, 20 g/l^−1^ glucose, and 20 g/l^−1^ agar, pH 7.0). The sequence of this strain’s rDNA internal transcribed space was submitted to the NCBI database under accession number KY910213.1. After 2 weeks of culture on PDA, the mycelia of F5 were collected using a sterile 5-mm diameter cork borer.

Raspberry seeds were sterilized in 75% alcohol solution for 30 s, washed three times with sterilized water, transferred to 2% sodium hypochlorite for 5 min, and then washed 5 times with sterilized water. The micropropagated seedlings of raspberry were transferred to a WPM medium. The seedlings were grown in the culture room at 28°C. After 3 months, aseptic raspberry seedlings were planted in a soil matrix (80% turfy soil and 20% vermiculite) and grown in a controlled growth room (25°C, 16/8 h, day/night, 1,600 lux). Twenty seedlings were inoculated with an F5 mycelium pellet obtained by liquid culture, and the remaining 20 were grown without F5 treatment as a control. The samples were labeled CK (control check, without inoculation), BI (presymbiotic phase, 3 weeks after inoculation), and UI (symbiotic phase, 5 weeks after inoculation). Sample roots were collected and separated into two parts. One part was used to check the F5 colonization rate, and the other part was flash frozen in liquid nitrogen and stored at −80°C for RNA extraction. To determine growth parameters, such as plant height, root length, fresh weight, and root activity (Peng et al., 2022), three biological replicates were performed to calculate the mean expression and standard deviation (SD).

### Analyses of F5 colonization in roots

Raspberry roots were fixed with formalin–acetic acid–alcohol (FAA) for 12 h. The roots were then treated with 10% KOH and heated for 1 h, followed by treatment with 1% HCl for 30 min at room temperature. Finally, the roots were stained with Trypan Blue (Liu et al., 2016). Dyed roots were detected after transparency with lactic acid and glycerin.

### Total RNA extraction, library construction, and small RNA sequencing

Total RNA was extracted from the collected root samples with TRIzol^®^ Reagent (Invitrogen, United States) according to the manufacturer’s instructions. Total RNA concentration and purity were measured by a NanoDrop 2000 (Thermo Scientific), and total RNA integration and quality were determined by agarose gel electrophoresis. RNA molecules that were less than 50 nt in length were enriched and ligated with proprietary adapters. The RNA samples ligated with adapters were reverse transcribed and amplified by PCR to produce sequencing libraries. The three sRNA libraries from raspberry roots were sequenced on an Illumina Hiseq 2,500 (Santiago, CA, United States) platform at Genedenovo Biotechnology Co., Ltd. (Guangzhou, China). The raw sequence data reported in this paper have been deposited in the Genome Sequence Archive (Genomics, Proteomics & Bioinformatics 2021) in National Genomics Data Center (Nucleic Acids Res 2022), China National Center for Bioinformation/Beijing Institute of Genomics, Chinese Academy of Sciences (GSA: CRA008006) that are publicly accessible at https://ngdc.cncb.ac.cn/gsa.

### Identification of known and novel miRNAs

High-quality reads were generated by removing the adaptor reads, low-quality sequences, and fragments of less than 18 nt or more than 30 nt from the raw reads. To classify the clean reads, they were aligned with the small RNAs and annotated to Rfam databases (Rfam 11.0, rfam. janelia.org) to identify and filter out the ribosomal RNA (rRNA), transport RNA (tRNA), nuclear small RNA (snRNA), nucleolar small RNA (snoRNA), and repeat sequences. After the removal of known miRNAs, the remaining sequences were used to identify novel miRNAs according to their hairpin structures predicted by Mireap_V0.2 software. Novel miRNAs were predicted from non-annotated sRNA sequences using miRDeep2 software (Friedländer et al., 2012). The basic criteria were used to screen for new candidate miRNAs (Zhang et al., 2015).

### Identification of F5-responsive miRNAs in raspberry

To identify F5 symbiosis-responsive miRNAs, raw miRNA counts from each sample were used to normalize miRNA expression. The fold changes in miRNA levels in treated samples were calculated relative to those in the control. The miRNAs with a fold-change in expression log^2^ (treatment/control) ≥ 2 and an adjusted *value of p* < 0.05 were considered differentially expressed and referred to as the F5 fungus-responsive miRNAs. Tool DEseq (version 1) was used to identify the differentially expressed miRNAs.

### Prediction of target genes

Based on the sequences of known and novel miRNAs, the putative target genes were predicted by the software psRNATarget algorithm with the following parameters: (1) maximum expectation is less than 5.0; (2) length for complementary scoring (hspsize) is shorter than 19; (3) range of central mismatch leading to translational inhibition is 10–11 nt. The mature sequences of all miRNAs were submitted to predict target genes; and (4) the minimum free energy (MFE) of the miRNA/target duplex should be greater than or equal to 74% of its miRNA bound to a perfect complement.

To elucidate the regulatory function and pathways involved in symbiosis, the predicted target genes were further annotated and enriched against GO using Blast2GO (version 2.8, accessed online)^1^ and against the KEGG pathway database using KAAS (KEGG Automatic Annotation Server, accessed online)^2^, respectively.

### Stem-loop qRT-PCR identification

Stem-loop primers were designed for qRT-PCR, as described by Chen et al. (2005). The primers are shown in Supplementary Table S1. To verify the relative expression of the miRNAs in the CK, BI, and UI groups, a reverse transcriptase reaction was performed using a HiFiScript cDNA Synthesis Kit (CW2569M, CWBIO, Beijing, China). The 20 ml mixture contained 0.5 mM each of dNTPs, stem-loop primers, 5 mg RNA template, 10 mM DTT, 200 U HiFiScript, and RNase-free water. Then, reverse transcription products were mixed with TB Green Premix Ex Taq (Tli RNaseH Plus, TaKaRa, Dalian, China) in a 96-well plate to start the real-time PCR reaction with a Roche LightCycler 480 II system using the following conditions: an initial denaturation step of 30 s at 95°C, 40 cycles of denaturation for 5 s at 95°C, 30 s at 60°C for annealing, and 30 s extension at 72°C. The relative expression of miRNAs was calculated based on the abundance of the reference gene U6 snRNA. The 2^–△△CT^ method was adopted to assess the relative miRNA expression from the qRT-PCR experiments (Livak and Schmittgen, 2001). Three biological replicates with three technical replicates were performed for each sample.

## Results

### Effects of F5 on the morphological and physiological of raspberry

**Figure 1:**
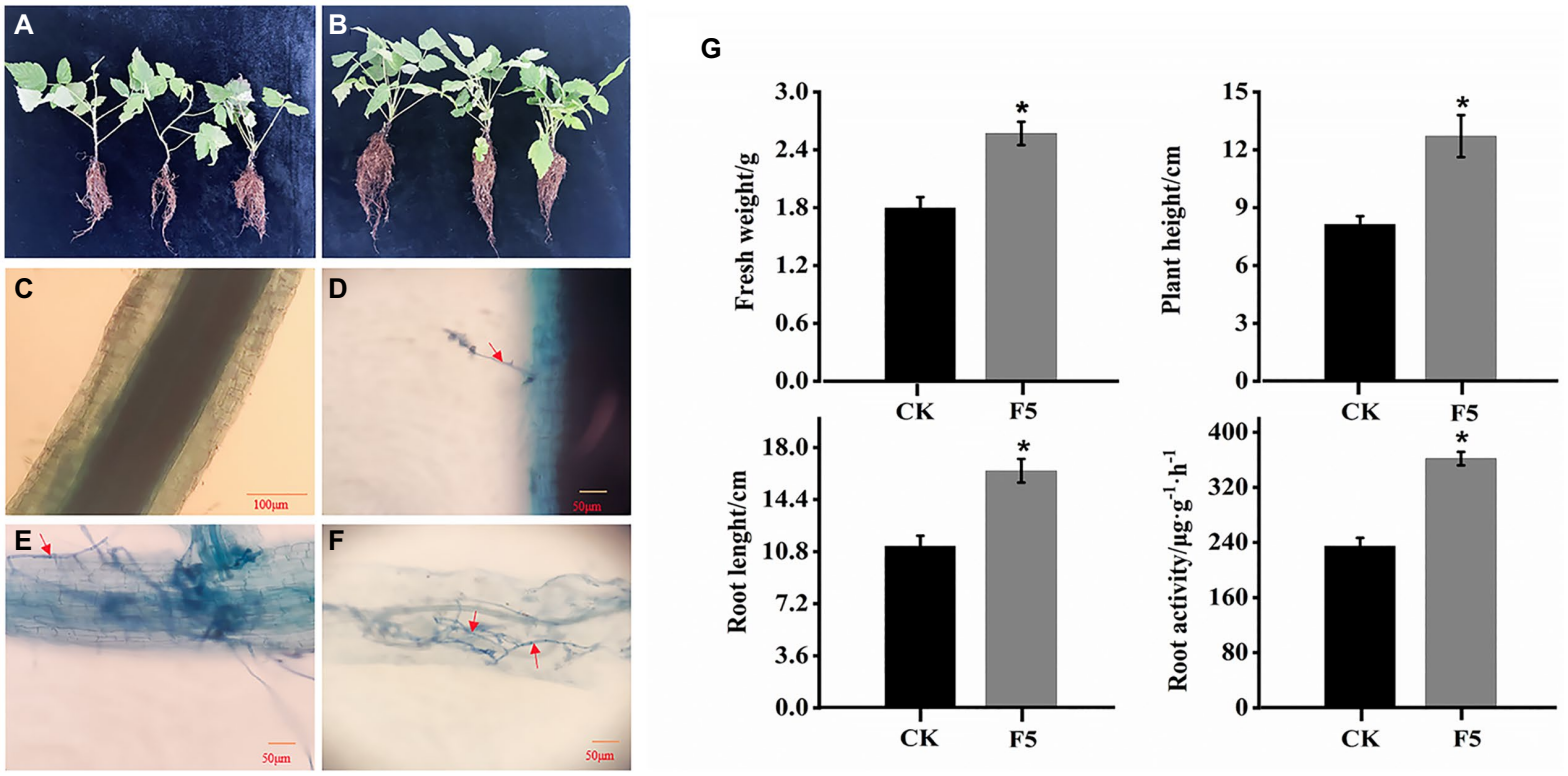
Effects of F5 on the growth of raspberry. **(A)** The growth of raspberry with F5. **(B)** The growth of raspberry with no microbial inoculation. **(C–F)** Dark septate endophytes (DSEs) stained with trypan blue in raspberry root tissue. **(C)** CK (control check, without inoculation). **(D)** BI (presymbiotic phase, 3 weeks after inoculation). Mycelia infecting raspberry cells. (**E,F)** UI (symbiotic phase, 5 weeks after inoculation), intercellular hyphae formed hyphal nets. The red arrow indicates the fungal mycelium. **(G)** Growth index measured using fresh weight, plant height, root length, and root activity measured in F5-treated plants and the control. Mean ± SD (*n* ≥ 20 seedlings per group). Mean ± SD (*n* ≥ 20 seedlings per group).

To evaluate the plant growth of DSE F5 on the growth of raspberry, we used F5 to inoculate *R. idaeus* seedlings. After 3 months, F5 inoculation had a dramatically positive effect on the fresh weight, plant height, root length, and root activity of raspberry. The F5-colonized raspberry seedlings exhibited more vigorous growth than the controls (Figures 1A,B). In particular, the root length of the F5-treated raspberry seedlings had significant advantages over that of the control. After 3 months of growth, F5-inoculated seedlings had a 43% greater fresh weight and 56% greater plant height than the control seedlings. Similarly, the root length and root activity of the F5-treated raspberry seedlings were 1.5 times greater than those of the controls (Figure 1G).

Microscopic observation confirmed that the fungal hyphae of F5 colonized the raspberry roots. Darkly pigmented hyphae were observed within the epidermal and cortical cells of F5-inoculated root hairs using trypan blue staining (Figures 1D–F). The features were identical to those previously described for DSEs. In contrast, the root cells of uninoculated seedlings were cleaner and showed no signs of infestation (Figure 1C).

### Overview of small RNAs from raspberry *via* high-throughput sequencing

**Figure 2:**
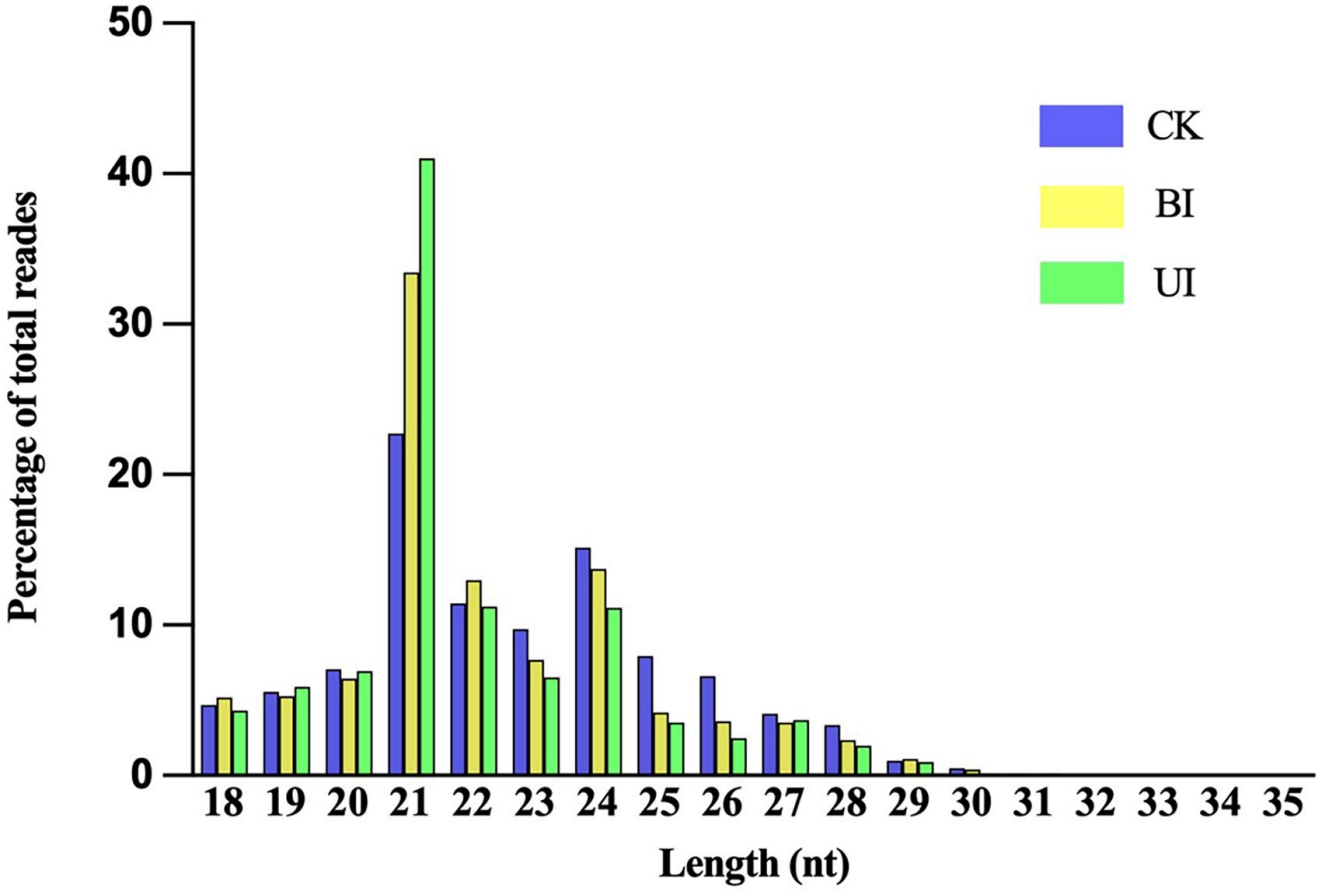
Distribution of clean reads with different sequence lengths according to their total reads in the CK (control check, without inoculation), BI (presymbiotic phase, 3 weeks after inoculation), and UI (symbiotic phase, 5 weeks after inoculation).

This study aimed to identify miRNAs responsive to symbiosis between raspberry and DSEs. A total of nine sRNA libraries comprising three samples (CK: control check; BI: the period before infection; UI: the period under infection) were generated using the Illumina sequencing platform. Three biological replicates were performed per treatment. Sequencing results showed that there were 15,157,505, 13,487,132, and 10,542,855 raw reads for CK, BI, and UI, respectively, while the clean reads comprised over 73.20% of all reads in the appropriate sizes of 18–35 nt without low-quality or substandard reads (Table 1). By analyzing the lengths of clean read sequences in samples from different periods, the sRNA sequence lengths were found to be mainly distributed between 18 and 30 nt, and mainly concentrated in 21–24 nt, with the largest number of sRNAs at 21 nt. The sequence length decreased sharply in abundance after 25 nt (Figure 2). The distribution of sRNAs in this study was similar to that in other dicotyledons (Rajagopalan et al., 2006; Moxon et al., 2008). Different lengths of sRNAs have different biological functions. This difference in abundance suggests that the expression of miRNAs in samples at different stages can be significantly different.

**Table 1:** Statistics of small RNA libraries analyzed by high-throughput sequencing.

Read data	CK	BI	UI
Raw reads	15,157,505	13,487,132	10,542,855
High-quality reads	15,067,606	13,408,253	10,484,440
3′ adapter null	98,171	54,666	258,887
Insert null	123,243	110,387	80,796
5′ adapter contaminants	24,446	29,250	11,594
PolyA	375	321	162
Clean reads	13,300,281	10,090,123	31,99,676

### Identification of known and novel miRNAs

**Figure 3:**
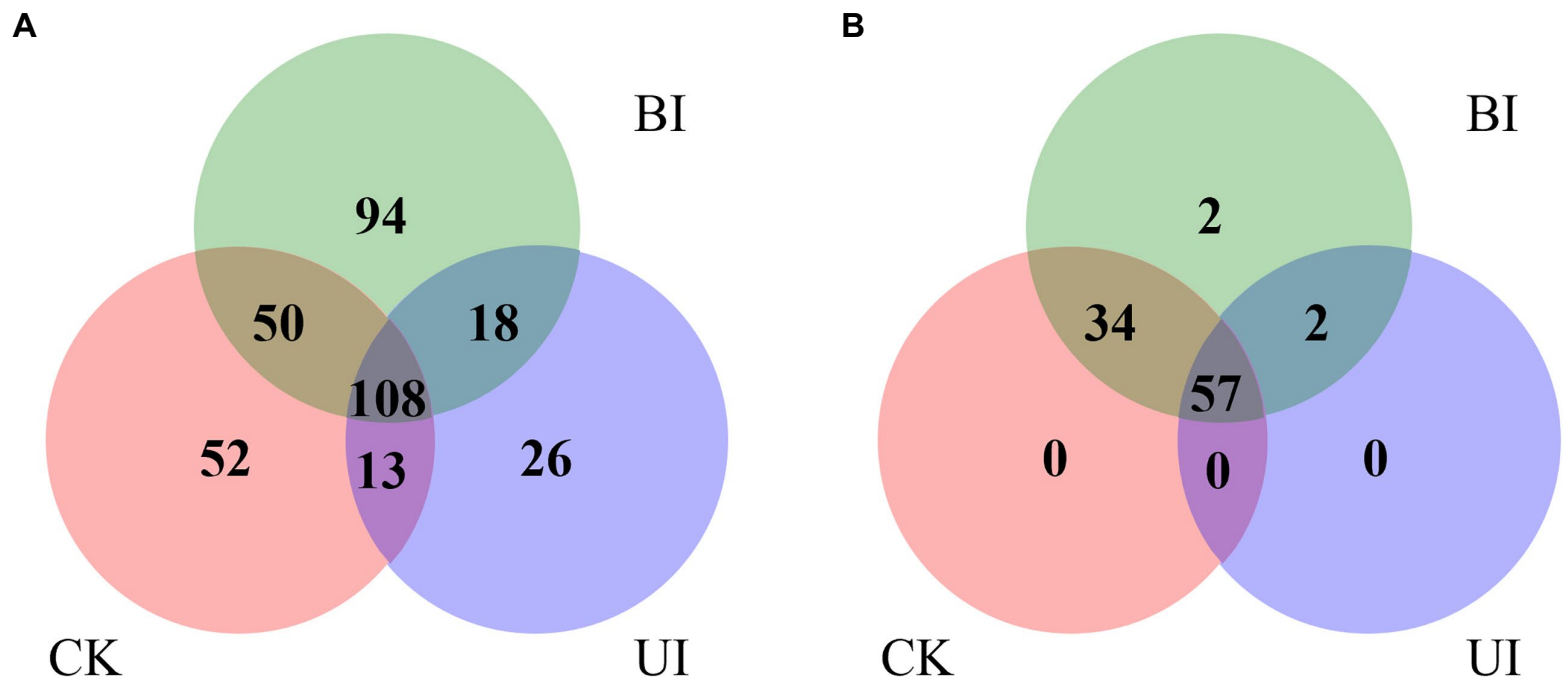
Venn diagram indicating the common and unique microRNAs in the control check (CK), the period before infection (BI), and the period under infection (UI) small RNA libraries. **(A)** Known miRNA distribution and **(B)** novel miRNA distribution.

After the sequencing data were filtered and quality-controlled, the clean reads were compared using the GenBank and Rfam databases to count those annotated to rRNA, tRNA, snRNA, and snoRNA. The proportion of rRNA was much higher than the proportion of other types, followed by tRNA and snRNA, and the lowest proportion was of snoRNA (Supplementary Table S2). The number of all annotated known and novel miRNAs distributed among the three comparisons of raspberry is shown in Figure 3. After removing small non-coding RNAs, such as rRNA, tRNA, snRNA, and snoRNA, the remaining valid sequences were compared with the mature sequences of plant miRNAs in the miRBase database.

**Figure 4:**
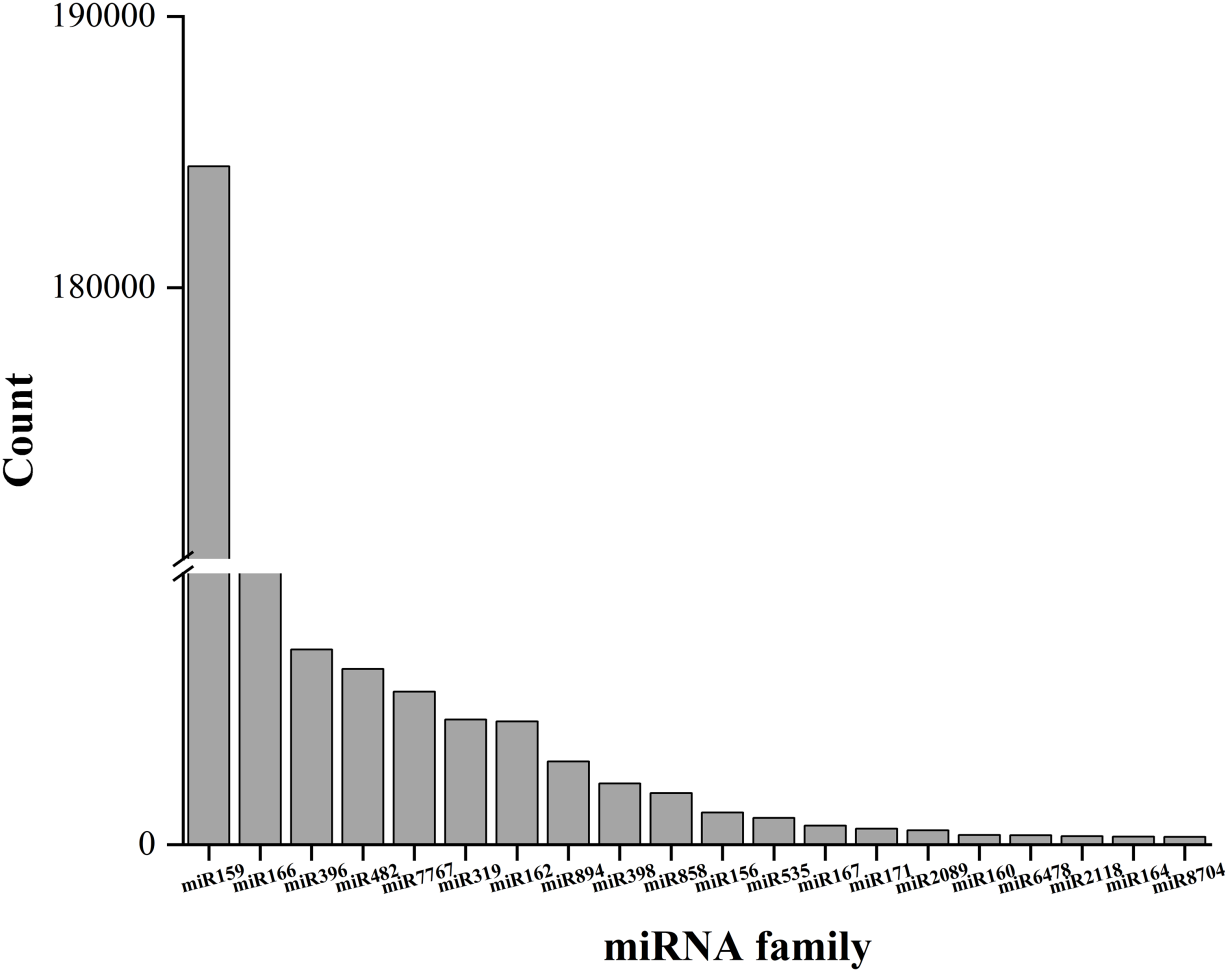
The top 20 miRNA families and their respective reads among the three comparisons. The miR159 family had the highest number of respective reads among all miRNA families.

A total of 361 known miRNAs were identified from three libraries. We describe the top 20 largest miRNA family reads among all miRNAs in Figure 4. Most miRNAs with high expression (*>* 100 in each group) were 21 nt in length. The hairpin structure of 50 miRNAs was successfully predicted using mireap_v0.2; the length of the miRNA hairpin ranged from 79 to 295 nt. The minimal folding free energy index (MFEI) of hairpin structures ranged from 0.51 to 1.18, and over 94% of the MFEIs of miRNAs were greater than 0.80. Detailed miRNA information is shown in Supplementary Table S3. These miRNA families were characterized by the presence of numerous miRNAs, as expected. Some miRNAs were only detected in a particular group, showing that certain miRNAs were group-specific (Supplementary Table S4). Most of these miRNA families were highly conserved in plants, such as miR159, miR166, and miR396. Among these, miR396 was the most conserved and existed in 46 plant species. This was followed by miR166 and miR159, which were present in 45 and 10 plant species, respectively. In general, the expression of known miRNAs in raspberry was not abundant; 85 known miRNAs were present in three comparisons at high abundance (> 100), and 276 known miRNAs were at low abundance (about 100). Most of these miRNAs have unknown functions in F5–raspberry symbiosis.

After removal of the known miRNAs and other noncoding RNAs, the remaining clean reads mapped to the raspberry transcriptome were used to predict novel miRNAs based on the secondary structure. Using Mireap_V0.2 software, 95 types of novel miRNAs were identified, and a list of novel miRNA sequences, hairpin MFEIs, precursor sequences, and structures are shown in Supplementary Table S5.

**Figure 5:**
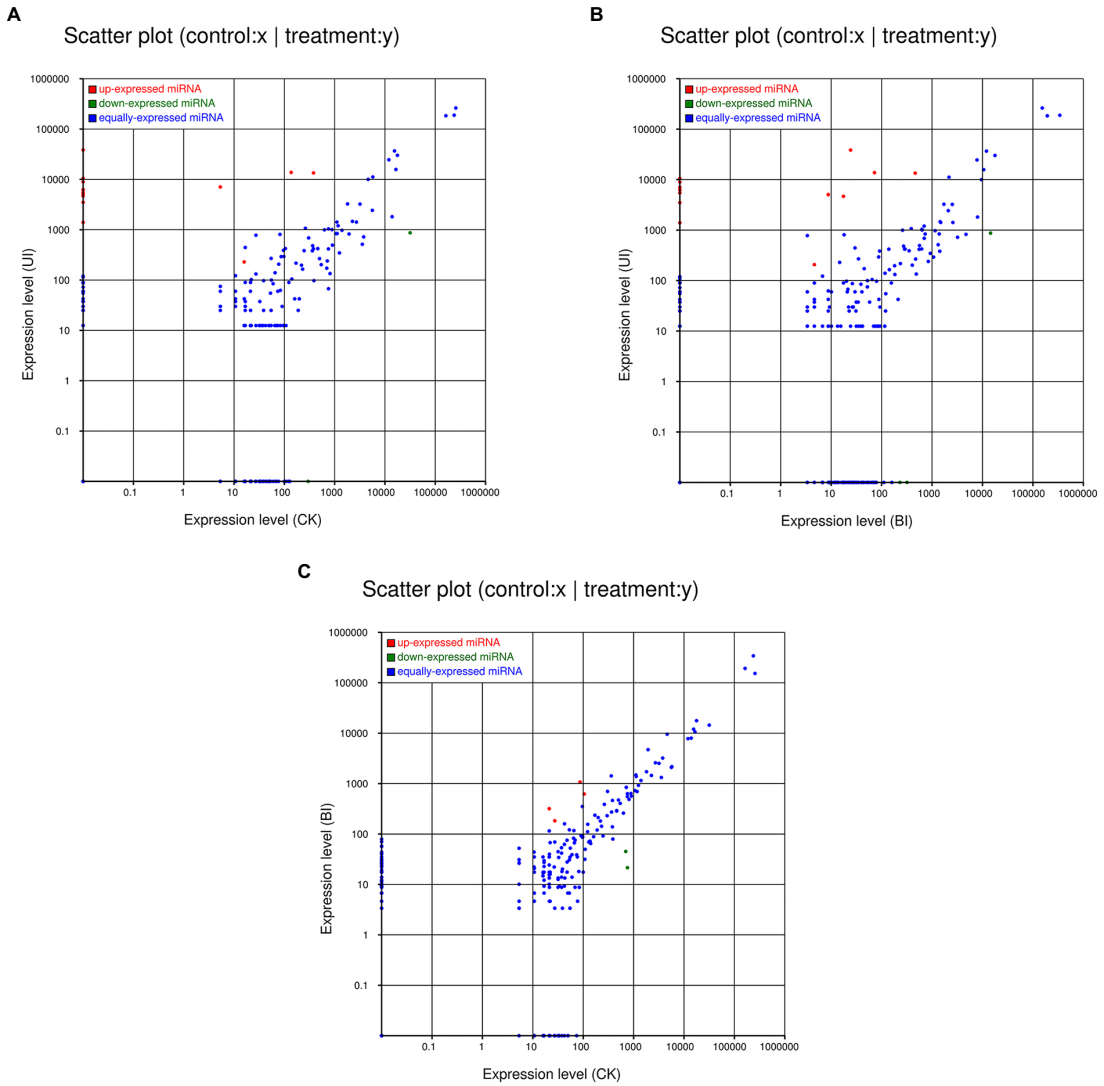
Comparisons of the different expression patterns of novel miRNAs between each pair of groups. Each point in the scatter plots represents one miRNA. The *x*-axis and *y*-axis individually show the expression levels between the two libraries. The red dots indicate a more abundant expression in the *y*-axis library (fold change *>*1, *p <* 0.05); the blue dots indicate equal expression between the two libraries (−1 *<* fold change *<*1, *p >* 0.05), and green dots indicate less enrichment in the *y*-axis library (fold change *<* −1, *p <* 0.05). **(A)** Expression in the CK (control check, without inoculation) vs. expression in the UI (symbiotic phase, 5 weeks after inoculation), **(B)** expression in the BI (presymbiotic phase, 3 weeks after inoculation) vs. expression in the UI (symbiotic phase, 5 weeks after inoculation), and **(C)** expression in the CK (control check, without inoculation) vs. expression in the BI (presymbiotic phase, 3 weeks after inoculation).

The expression levels of the novel miRNAs among the CK, BI, and UI libraries were compared (Figure 5). In CK, BI, and UI, 81, 85, and 59 novel miRNAs, respectively, were identified, showing that the number of novel miRNAs was much smaller than the number of known miRNAs. Based on Figure 5, we concluded that most of the novel miRNAs were equally expressed among the three libraries, while several of the differentially expressed novel miRNAs deserve further attention to discern their functions. For example, novel miRNAs, such as novel-m0090-5p, novel-m0091-3p, novel-m0094-3p, and novel-m0095-3p, were highly expressed only in the BI group; novel-m0072-5p, novel-m0073-5p, novel-m0076-5p, and novel-m0078-5p were highly expressed in the CK and BI groups but not in the UI group. Detailed information on the predicted novel miRNAs is listed in Supplementary Table S6.

### Identification of putative F5-responsive miRNAs in raspberry

To identify the differentially expressed miRNAs related to F5 in raspberry, we detected miRNA expression levels in nine sRNA libraries (including three biological duplicates). The relative expression of miRNAs from different libraries was calculated using log_2_, with a fold change ≥ 2 and *p*-value < 0.05 as screening conditions. The results showed that 13, 22, and 21 miRNAs were differentially expressed in CK vs. BI (6 upregulated and 7 downregulated), BI vs. UI (15 upregulated and 7 downregulated), and CK vs. UI (16 upregulated and 5 downregulated), respectively (Supplementary Tables S7–S9).

Differential expression analysis revealed potential key regulatory miRNAs during F5 infestation in raspberry. A total of 25 known miRNAs were differentially expressed in the three comparisons. A total of 6 (4 upregulated and 2 downregulated) differentially expressed miRNAs were found in the CK vs. BI comparison group. In total, 15 and 16 known miRNAs were upregulated in the BI vs. UI and CK vs. UI comparison groups, respectively, of which 14 miRNAs were identical. The log2 of miR171-z, miR396, miR11532-z, miR11561-z, and miR1311-x exceeded 19.05, while miR156-y and miR7767-x were downregulated in both groups, indicating that the symbiosis-related miRNAs formed *in vivo* by F5 remained largely unchanged at the middle and late stages of raspberry infestation, acting together to promote symbiosis formation.

Additionally, we analyzed novel miRNA expression patterns. Fourteen novel miRNAs were differentially expressed, with a total of 7 (2 upregulated and 5 downregulated) differentially expressed miRNAs in CK vs. BI. A total of 4 miRNAs were downregulated in BI vs. UI, and 4 (1 upregulated and 3 downregulated) differentially expressed miRNAs were identified in CK vs. UI. Among them, novel-m0002-3p and novel-m0021-3p were downregulated in both the BI vs. UI and CK vs. UI comparison groups. Overall, the upregulated miRNAs were more abundant than the downregulated miRNAs in plants during the F5 infestation of raspberry. These results suggest that both known and novel miRNAs may play specific and important roles in the response of raspberry to DSE infestation.

### Prediction and functional analysis of putative target genes of DSE-responsive miRNAs

miRNAs perform their functions by regulating the expression of their target genes or repressing their translation. Therefore, the prediction and identification of target genes is an important part of understanding the various biological functions of miRNAs. We performed target gene prediction using psRNATarget, and all miRNAs were predicted to have corresponding target genes. Among the identified miRNA-target pairs, target gene prediction was generally not a simple one-to-one relationship but rather a mismatched relationship. A single miRNA may regulate multiple target genes. In this article, 24,112 target genes were predicted for known miRNAs, while 1,367 target genes were obtained for novel miRNAs (Supplementary Tables S10, S11).

**Figure 6:**
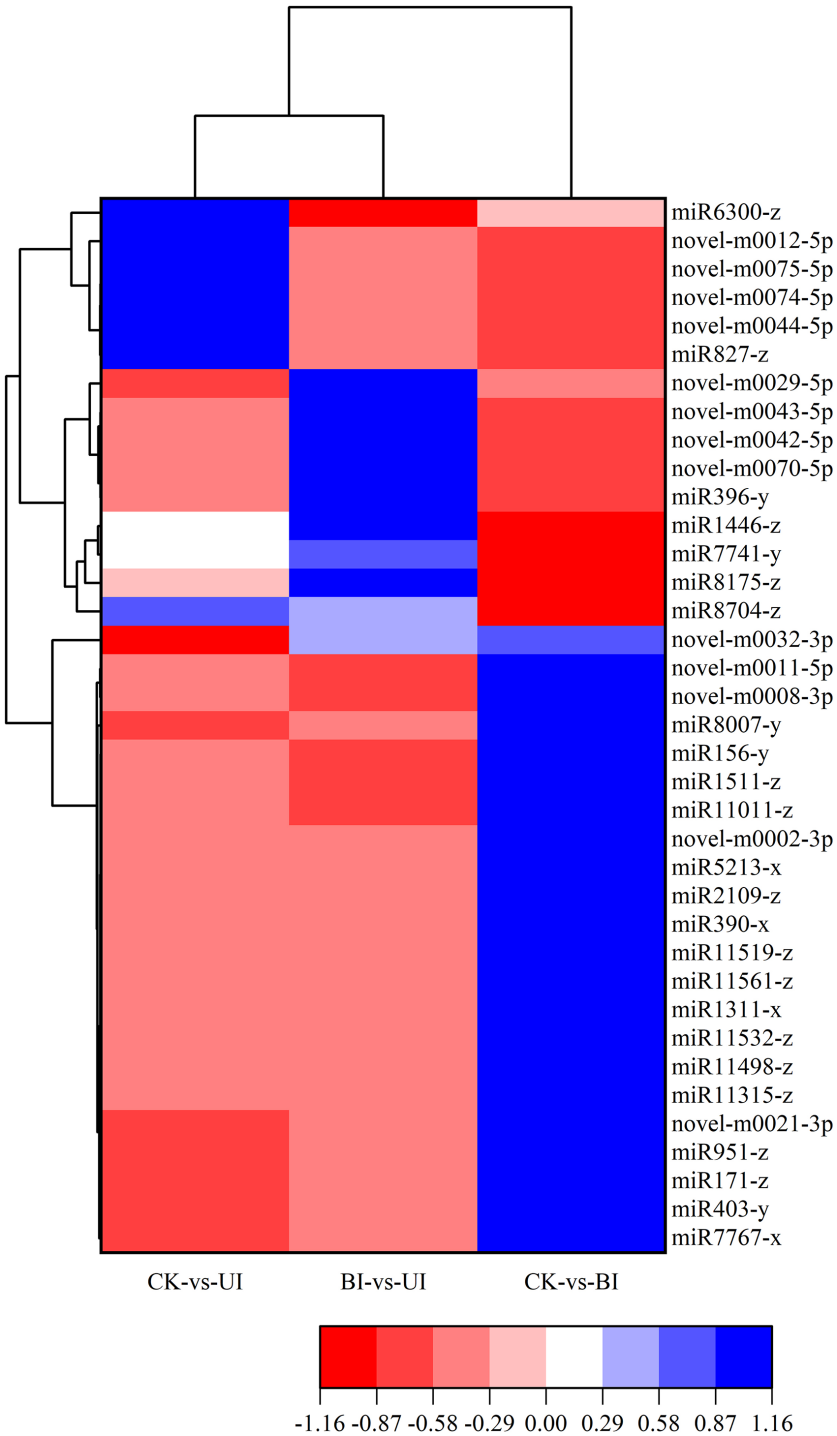
Heatmap of the differentially expressed miRNAs. The color scale represents the relative expression level of differentially expressed miRNAs.

There was significant differential miRNA expression between CK, BI and UI (Figure 6). To further investigate the role of responsive symbiotic miRNAs in F5–raspberry symbiosis, we selected miRNAs differentially expressed in F5-infected raspberries for target gene prediction in the middle and late stages. A total of 561 target genes targeted by 37 differentially expressed miRNAs were identified. The annotations of some of the target genes responding to the co-expressed miRNAs are shown in Table 2. A large proportion of these target genes had specific or presumed functions, and these target genes were involved in the regulation of diverse metabolic processes.

**Table 2:** Prediction of target genes in response to F5 symbiotic miRNA in raspberry.

miRNA	Description of target gene	References
miR11011-z	TMV resistance protein N-like, probable disease resistance protein At4g272ss20	Guo et al. (2016), Dang et al. (2021)
miR11315-z	F-box protein CPR30-like	Gou et al. (2012)
miR11561-z	LRR receptor-like serine/threonine-protein kinase At3g47570	Sun et al. (2015)
miR396-y	GRF transcription factors; ceramidasegenes leaf and cotyledon development	Krebs et al. (2007)
miR1511-z	40S ribosomal protein S26E, glutamate receptor 2.8-like, chitinase-like protein 1	Tartar and Boucias (2004), Berek et al. (2009)
miR171-z	toll/interleukin-1 receptor-like protein, Scarecrow-like protein	Ding et al. (2012), Ru et al. (2015)
miR390-x	high affinity inorganic phosphate transporter, leucine-rich repeat receptor, Auxin response factor	Preuss et al. (2011), Yamaguchi et al. (2006)
miR403-y	DNA replication complex GINS protein SLD5	Joshi et al. (2016)
miR5213-x	BURP domain-containing protein 5-like, calcium-dependent protein kinase 2-like	Boudsocq et al. (2012), Dinh and Kang (2017)
miR6300-z	RING finger protein ETP1-like, zinc finger BED domain-containing protein RICESLEEPER 2-like	Shen et al. (2014)
miR951-z	Growth-regulating factor	Kuijt et al. (2014)
miR8007-y	domain-containing protein, polyubiquitin 3	Christensen et al. (1992)
miR156-y	60S ribosomal protein L9, Auxin response factor	Yu et al. (2012)
miR7767-x	nodulation protein H, cell wall-associated hydrolase	Bloemberg et al. (2010), Liu et al. (2017)
miR827-z	cryptochrome1	Keller et al. (2011)
novel-m0008-3p	sucrose synthase 2	Zrenner et al. (2010)
novel-m0029-5p	senescence-associated protein, heme-binding protein	Kasaras et al. (2012), Shanmugabalaji et al. (2020)
novel-m0011-5p	metallothiol transferases FosB	Thompson et al. (2015)
novel-m0021-3p	DUF538 family protein	Takahashi et al. (2013)
novel-m0032-3p	protein-lysine methyltransferase	Sheng et al. (2016)

To further analyze the possible regulatory pathways involved in miRNA target genes, GO and KEGG functional enrichment analyses were performed for all target genes in this study. GO enrichment analysis showed that many target genes were associated with biological process (GO:0008150), metabolic process (GO:0008152), cellular process (GO:0009987), organic substance metabolic process (GO:0071704), and cellular metabolic process (GO:0044237) in the biological process. For cellular components, the main terms were cell (GO:0005623), cell part (GO:0044464), and membrane (GO:0016020), and important terms for molecular functions were binding (GO:0005488), catalytic activity (GO:0003824), and transferase activity (GO:0016740; Figure 7).

**Figure 7:**
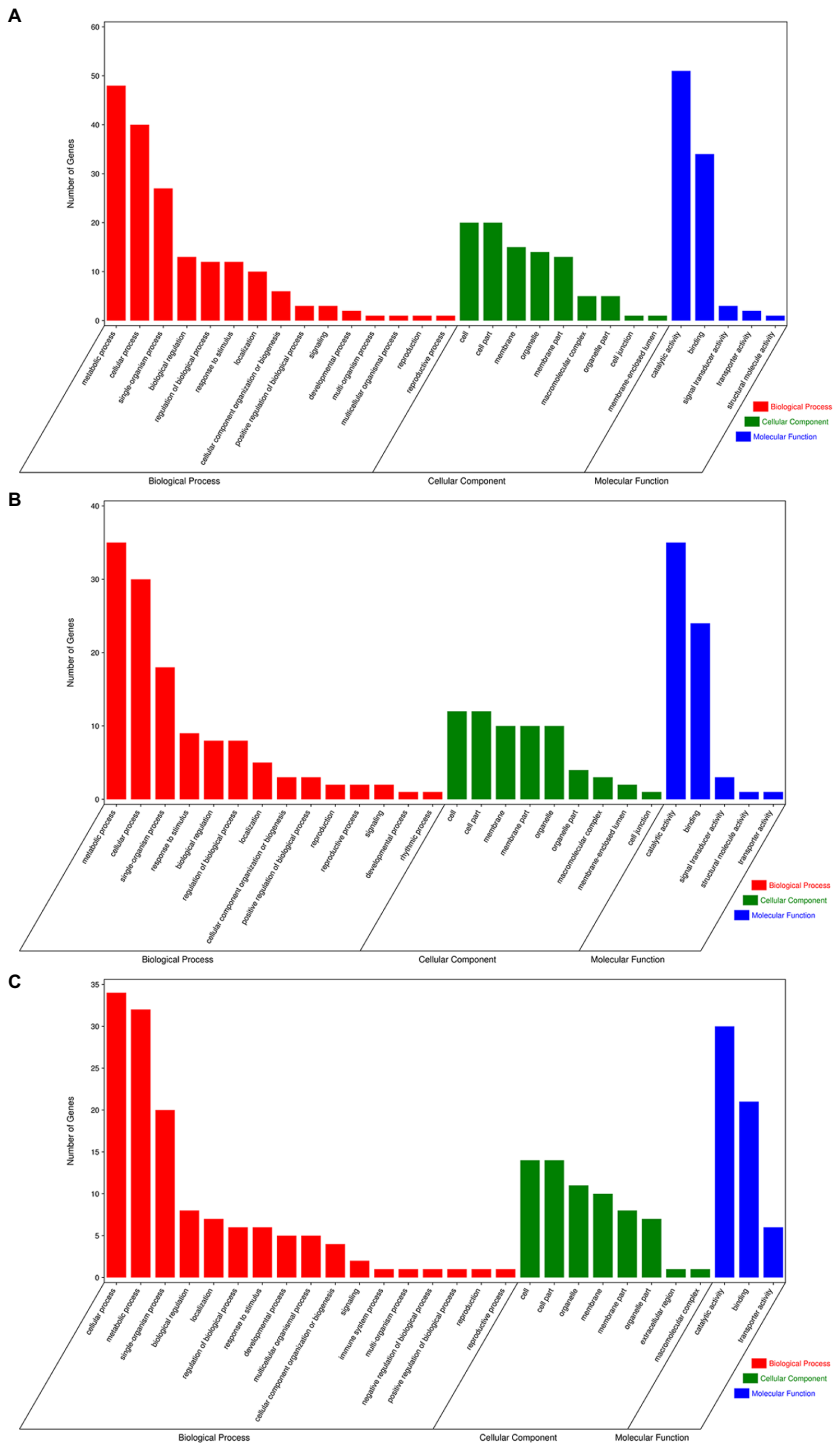
Gene ontology of the predicted targets for differentially expressed miRNAs. **(A)** GO terms for CK vs. UI, **(B)** BI vs. UI, and **(C)** CK vs. BI.

**Figure 8:**
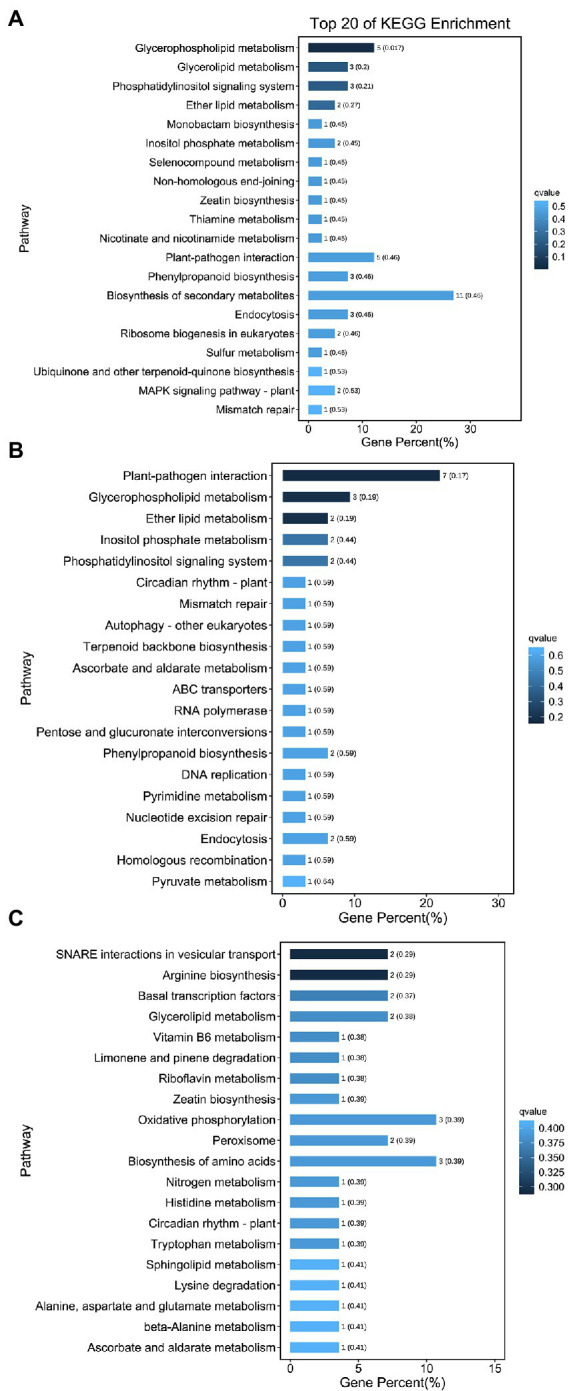
Kyoto Encyclopedia of Genes and Genomes (KEGG) analysis of the 20 most enriched pathways from the roots. **(A)** KEGG analysis for CK vs. UI, **(B)** BI vs. UI, and **(C)** CK vs. BI. The coloring of the *q*-values indicates the significance of the enrichment factors. The Y-axis shows the names of the enriched pathways. The X-axis represents the percentage of enriched genes.

Kyoto Encyclopedia of Genes and Genomes functional enrichment showed that the pathways with the highest enrichment of target genes were Biosynthesis of secondary metabolites (ko01110), Plant–pathogen interaction (ko04626), Glycerophospholipid metabolism (ko00564), Oxidative phosphorylation (ko00190), and Biosynthesis of amino acids (ko01230; Figure 8). Notably, five genes were annotated to the plant–pathogen interaction pathway, namely Unigene0062736 (CPK2), Unigene0014710 (MEKK1), Unigene0047782 (EFR), Unigene0017630 (CML38), and Unigene0020119 (At4g27190). All five genes are involved in plant immune responses, which play an important role in symbiosis formation between plants and rhizosphere microorganisms and are usually specifically repressed from rhizosphere microorganisms, thus promoting the invasion and proliferation of rhizosphere microorganisms (Cao et al., 2017). In this study, five genes were targeted by miR11011-z, miR2109-z, miR8007-y, and miR390-x. All four miRNAs were upregulated in symbiosis; therefore, it was hypothesized that miRNAs may affect F5–raspberry symbiosis by regulating plant immune responses.

### Quantitative PCR expression analysis

**Figure 9:**
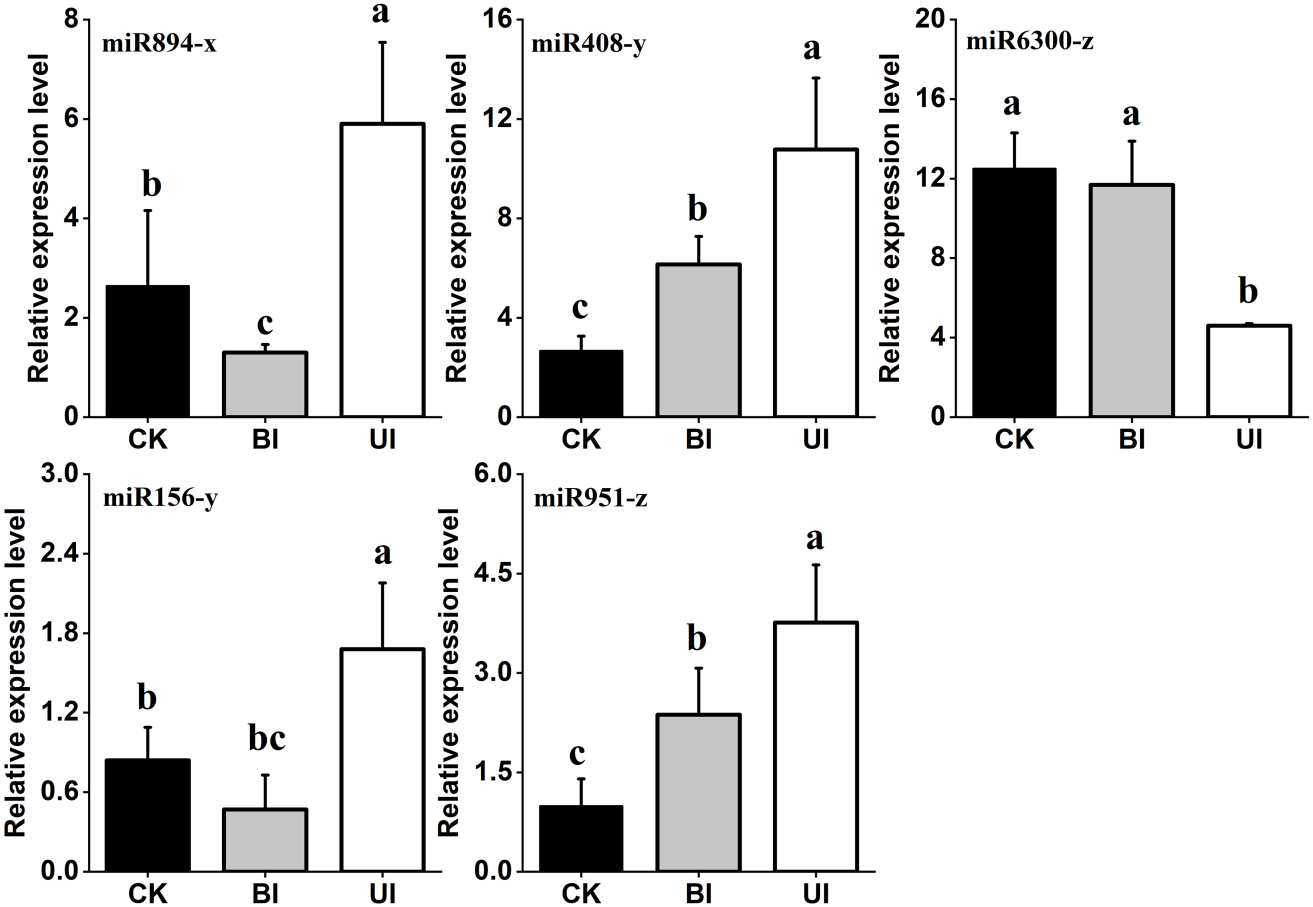
Quantitative reverse transcription polymerase chain reaction (qRT-PCR) validation of the expression profiles of five miRNAs. The expression levels of miRNAs were normalized to the level of U6 snRNA. Fold changes in the expression levels of miRNAs were estimated by the 2^–△△CT^ method relative to the levels in the roots. Data are reported as the mean ± standard error (SE) for three independent experiments. CK (control check, without inoculation), BI (presymbiotic phase, 3 weeks after inoculation), and UI (symbiotic phase, 5 weeks after inoculation).

Based on the identification of symbiotic miRNAs, quantitative real-time (qRT)-PCR analysis was used to verify the expression patterns of five representative miRNAs. Although the expression levels of each miRNA differed between the high-throughput sequencing and qRT-PCR analyses, the overall trend was consistent, indicating that the relative expression level identified by the sequencing data was reliable and suitable for further analyses (Figure 9).

## Discussion

Raspberry has high economic, nutritional, and medicinal value and is known as “the king of fruits” in the international market (Jagtap and Bapat, 2015). It is a “third-generation fruit” that is currently popular throughout the world. In recent years, the global raspberry planting area has increased, and the traditional root-tiller division, cutting propagation, and *in vitro* culture cannot meet the increasing demand due to slow propagation speed (Georgieva et al., 2016; Vadim et al., 2018). Data have shown that raspberry can form beneficial symbioses with various soil fungi, which can play an important role in promoting the absorption of nutrients and water and in improving environmental stress resistance (Correia et al., 2019; Hilje-Rodríguez and Molina-Bravo, 2020). Therefore, in this study, a dark septate endophyte (DSE) named F5, which was preserved in the laboratory, was selected to inoculate raspberry plants, and after 3 months of inoculation, it significantly improved plant growth.

Numerous studies have shown that DSEs have positive effects on host plants (Monica et al., 2015; Hou et al., 2020). However, it is controversial whether all species of DSES have a positive effect on plants. Newsham discussed 18 studies using meta-analysis to investigate the effect of DSE on plants. The results showed that DSE had a positive effect on plants, increasing total biomass as well as stem nitrogen and phosphorus content (Newsham, 2011). In contrast, another meta-analysis showed that inoculating plants with root fungal endophytes (including DSEs) had negative or neutral effects on plant biomass and nitrogen content (Mayerhofer et al., 2013). F5 in this experiment is the strain screened and isolated by ourselves, which can form a good symbiotic relationship with blueberry and raspberry, both of which can promote the growth of host plants and enhance the absorption of nitrogen and phosphorus in plants. To investigate the reasons for the increased growth of raspberry after F5 inoculation, we compared miRNAs from non-inoculated, 3-week inoculated, and 5-week inoculated raspberry plants by high-throughput sequencing and bioinformatics analysis. We screened for symbiotic-regulated miRNAs and predicted their target genes to further reveal the molecular mechanism of F5–raspberry symbiosis.

Analysis of high-throughput sequencing results showed that the sRNA sequence lengths were mainly distributed in 21–24 nt, with the largest number of sRNAs at 21 nt. The distribution of sRNAs in this study was similar to that of other dicotyledons (Rajagopalan et al., 2006; Moxon et al., 2008). A total of 361 known miRNAs and 95 novel miRNAs were identified by bioinformatics analysis. It has been reported that the expression of known miRNAs in most plants is higher than that of novel miRNAs (Yang et al., 2013; Dan et al., 2018). Among them, the expression of most miRNAs was very low, indicating that sRNA sequencing technology is an effective strategy for the comprehensive identification of miRNAs in plants. Furthermore, we predicted the sequences, targets, and secondary structures of these miRNAs in different groups to determine their functions and interference mechanisms during F5–raspberry symbiosis.

In this study, approximately 34 highly conserved miRNA families (total reads *>* 100) were detected; miR159 had the most reads, followed by miR166 and miR396. Surprisingly, the highly expressed miRNAs in raspberry showed similar expression patterns to those in other plants (Baksa et al., 2015; Dan et al., 2018; Zhou et al., 2018). The results of this study were also consistent with the fact that miRNAs are highly conserved in plants. These highly expressed miRNAs may play an important regulatory role in plant growth, as well as in F5–raspberry symbiosis.

The regulation of MYB by miR159 plays an important role in plant growth, flowering induction, male reproduction, flower organ development, plant fertility, fruit development, and stress response. Currently, studies based on the miR159-MYB pathway have been conducted in-depth in *Arabidopsis thaliana*. Progress has also been made in rice, cucumber, grape, barley, strawberry, tobacco, and other plants (Allen et al., 2005; Alonso-Peral et al., 2010). Some studies have shown that miR166 can participate in the growth and development of plant roots by cutting the target mRNA of the *HDZIP III* gene (Gutierrez et al., 2009; Xue et al., 2017). Under drought stress, plants can effectively resist and adapt to drought stress by increasing root growth, changing root morphological structures, and reducing the damage to plants caused by drought stress (Peng et al., 2013). miR396 targets mRNA coding for growth-regulating factor (GRF) transcription factors, rhodenase-like protein, and kinesin-like protein B (Kong et al., 2022). Some members of the miR482 family can produce secondary siRNAs through targeted cleavage of nucleotide binding site-leucine rich repeat-like (NBS-LRR-like) genes, thereby silencing and regulating a variety of genes related to plant disease resistance, including NBS-LRR-like genes (Sagi et al., 2017).

Although deep sequencing has been widely used to explore miRNAs, reports of symbiosis-associated miRNAs are still scarce. Symbiosis-related miRNAs have been studied mainly in the legume–rhizobia system, as well as in arbuscular mycorrhizal fungi–plant interactions (Gu et al., 2010; Bazin et al., 2012). miRNAs, which do not encode proteins themselves, participate in physiological processes, such as organogenesis, growth and development, hormone secretion, signal transduction, and stress response of eukaryotes by regulating target genes with corresponding functions. Target gene prediction and identification are prerequisites for in-depth studies of the functions of miRNAs in various biological processes in plants. In this study, 37 miRNAs responding to F5–raspberry symbiosis were identified by testing three root samples. The target genes of miRNAs in all symbiotic processes were predicted using psRNATarget. Among the miRNAs whose expression was repressed, miR171-h was highly induced by symbiosis in raspberry roots. Currently, studies on miR171-h family members, such as miR171-h and miR171-z, have mainly focused on mycorrhizal symbiosis. Lauressergues et al., 2012 analyzed the interaction between miR171-h and its target genes. When mycorrhizal fungi colonized the plant, miR171-h expression was upregulated in the root extension region. Mycorrhizal colonization was significantly reduced after miR171-h overexpression, while it was significantly increased by the expression of mutant target genes resistant to miR171-h shearing. It has been hypothesized that mycorrhizal factors (Myc-LCOs) induce mycorrhizal invasion, and the plant negatively regulates the target genes through miRNA to prevent excessive fungal colonization. In this study, miR171-z was also found to target the NSP2 gene in raspberry by target gene prediction. This indicates that the miR171 family is highly conserved across species by regulating NSP2 genes to suppress fungal–plant symbiosis.

miR396 has been reported to regulate root division and mycorrhizal colonization in *M. truncatula*. Overexpression of the miR396b precursor caused downregulation of six growth regulator factors (Mt-GRF) and two transcription factor bHLH79-like genes, as well as a decrease in mycorrhizal number and root growth, whereas miR36 inhibition increased the mycorrhizal number and root mass (Bazin et al., 2013). The study also found that miR396 was highly induced by symbiosis in raspberry, implying that miR396 regulates fungi–plant symbiosis by targeting GRFs that are highly conserved across species. These results demonstrate the conserved nature of symbiosis regulation in plants and further prove the reliability of the data in the present study. The target genes of miR156 regulate auxin biosynthesis and are involved in the regulation of cell division, organ growth, lateral root and adventitious root development, and signal transduction (Ellis et al., 2005; Nguyen et al., 2015). The target genes of miR827 were OsSPX-MFS1 and OsSPX-MFS2, which encode SPX-MFS proteins predicted to be implicated in phosphate sensing or transport (Lin et al., 2010).

The identification of fungal–plant symbiotic miRNAs and target gene prediction have been conducted in soybean (Subramanian et al., 2008), alfalfa (Fan et al., 2014), and tomato (Gu et al., 2010), which was important for understanding the regulatory mechanism of F5–raspberry symbiosis from GO and KEGG functional pathway enrichment results. The analysis of raspberry root miRNAs and their target genes under symbiotic conditions revealed that the expression of conserved miR399 and miR398 was increased, while miR396, miR827, miR171, and other conserved miRNAs were repressed by symbiosis. Further functional prediction analysis of miRNA-mediated target genes revealed that most of these target genes are transcription factors or functional proteins involved in the regulation of basic metabolism and abiotic stress for plant growth and development. In addition, GO and KEGG enrichment showed that they are mainly involved in biological processes, cellular metabolic processes, and regulatory mechanisms, including secondary metabolite biosynthesis, plant–pathogen interactions, and phytohormone signaling pathways. These findings provide valuable information for understanding the regulatory mechanisms of F5–raspberry symbiosis involving miRNAs.

In conclusion, 361 known miRNAs and 95 novel miRNAs were identified in this systematic analysis of the F5–raspberry root system. Thirty-seven miRNAs responding to F5–raspberry symbiosis were identified, and the functions of these miRNAs or target genes need to be further validated. This study not only demonstrated that miRNA-mediated post-transcriptional regulation of F5–raspberry symbiosis is conserved in plants but also provided candidate miRNAs for studying the molecular mechanism of F5–raspberry symbiosis.

## Data availability statement

The original contributions presented in the study are included in the article/Supplementary material, further inquiries can be directed to the corresponding authors.

## Author contributions

ZY, HZ, and JJ performed the experiments and analyzed the data. ZY, HZ, and LY detected the expression of miRNAs. ZY, HY, and LL wrote the manuscript. HY and LL designed the experiments and provided lab space and funding for this work. All authors read and approved the manuscript.

## Funding

The work was supported by the Fundamental Research Funds for the Central Universities (No. 2572021AW18), the National Natural Science Foundation of China (31971694 and 32071806), and the Natural Science Foundation of Heilongjiang Province (LH2020C101).

## Conflict of interest

The authors declare that the research was conducted in the absence of any commercial or financial relationships that could be construed as a potential conflict of interest.

## Publisher’s note

All claims expressed in this article are solely those of the authors and do not necessarily represent those of their affiliated organizations, or those of the publisher, the editors and the reviewers. Any product that may be evaluated in this article, or claim that may be made by its manufacturer, is not guaranteed or endorsed by the publisher.

## Footnotes

^1^ http://www.blast2go.com

^2^ https://www.genome.jp/kegg/kaas/
